# Surface Modification of Direct-Current and Radio-Frequency Oxygen Plasma Treatments Enhance Cell Biocompatibility

**DOI:** 10.3390/ma10111223

**Published:** 2017-10-25

**Authors:** Wan-Ching Chou, Rex C.-C. Wang, Cheng Liu, Chyun-Yu Yang, Tzer-Min Lee

**Affiliations:** 1 Institute of Manufacturing Information and Systems, National Cheng Kung University, Tainan 701, Taiwan; jinjaychou@gmail.com (W.-C.C.); ccwang@mail.ncku.edu.tw (R.C.-C.W.); 2 Hyperbaric Oxygen Therapy Center and Division of Plastic Surgery, Chi Mei Medical Center, Tainan 710, Taiwan; orangeliu@gmail.com; 3 Department of Electrical Engineering, Southern Taiwan University of Science and Technology, Tainan 710, Taiwan; 4 Department of Orthopedics, National Cheng Kung University Hospital, Tainan 704, Taiwan; cyyang@mail.ncku.edu.tw; 5 Institute of Oral Medicine, National Cheng Kung University Medical College, Tainan 704, Taiwan; 6 School of Dentistry, Kaohsiung Medical University, Kaohsiung 807, Taiwan; 7 Department of Dentistry, Kaohsiung Medical University Hospital, Kaohsiung 807, Taiwan

**Keywords:** sand-blasting and acid etching (SLA), Direct-Current (DC) plasma treatment, Radio-Frequency (RF) plasma treatment, amphoteric OH, protein adsorption, cell response

## Abstract

The sand-blasting and acid etching (SLA) method can fabricate a rough topography for mechanical fixation and long-term stability of titanium implant, but can not achieve early bone healing. This study used two kinds of plasma treatments (Direct-Current and Radio-Frequency plasma) to modify the SLA-treated surface. The modification of plasma treatments creates respective power range and different content functional OH groups. The results show that the plasma treatments do not change the micron scale topography, and plasma-treated specimens presented super hydrophilicity. The X-ray photoelectron spectroscopy (XPS)-examined result showed that the functional OH content of the RF plasma-treated group was higher than the control (SLA) and DC treatment groups. The biological responses (protein adsorption, cell attachment, cell proliferation, and differentiation) promoted after plasma treatments, and the cell responses, have correlated to the total content of amphoteric OH groups. The experimental results indicated that plasma treatments can create functional OH groups on SLA-treated specimens, and the RF plasma-treated SLA implant thus has potential for achievement of bone healing in early stage of implantation.

## 1. Introduction

Titanium is widely used in orthopedics and dental implants because of its good properties, including excellent corrosion resistance and good biocompatibility [1,2]. However, since a titanium surface is bioinert, the bone/implant interface does not initially bond well after implantation [3]. Many studies have attempted to apply surface treatment to the modify roughness, topography, and chemistry of titanium [4,5,6,7,8]. The sand-blasting and acid etching (SLA) method achieves micron and submicron scale topography for implant fixation. Previous clinical studies have reported the characteristics of SLA implants, which can restore bone defects after a healing period of 6–8 weeks [9,10]. Although SLA treatment for bone fixation does not alter the surface composition of titanium, SLA-treated surfaces belong to hydrophobic properties. The hydrophobicity of SLA surfaces is unfavorable for protein adsorption and cell affinity [11]. Zhao et al. used nitrogen protection to diminish hydrocarbon contamination and enhance hydrophilic property of SLA-treated titanium [12]. The results indicated that hydrophilic surface was shown to greatly induce osteoblast cell differentiation. Klein et al. used the same procedure to prepare the modified-SLA specimens with hydrophilic property [13]. They found that modified-SLA surface-promoted cell adhesion and exhibited highest levels of Integrin β1, Integrin αv, Runx-2, Collagen, Alkaline Phosphatase, osteocalcin of osteoblastic cell. However, Bacakova et al. indicated that highly hydrophilic surfaces are possible to prevent the adsorption of proteins or molecules [14]. 

In clinical use, the oxide film of titanium may play an important role in biological responses. The oxide layer of TiO_2_ is known to have two types of hydroxyl groups, basic and acidic OH, attached to the metal by chemisorption [15]. The functional OH groups tend to simultaneously act as anion and cation exchange sites. These sites could provide electrostatic bonding for adsorption of lipoprotein, glycolipids, proteoglycans, and collagen filaments. In our previous studies, different kinds of surface treatment were used to modify properties of oxide film on smooth titanium alloy [15,16,17,18]. The results indicated that the functional OH group of oxide film would influence protein adsorption, cell morphology, cell adhesion strength, and cell proliferation. It is very interesting that the functional OH group could also play an important role in biological responses of micron and submicron scale rough surfaces.

Plasma treatments are effective surface modification techniques, as they can modify physicochemical properties, such as hydrophilicity, increasing surface energy, and improving biocompatibility [19]. Plasma treatments have been used in biomaterial applications, such as plasma-induced grafting, surface cross-linking, eliminating surface contamination, and promoting cell adhesion. Moreover, surface properties, such as surface energy and functional groups, controls protein adsorption, as well as cell adhesion and differentiation. Canullo et al. used plasma treatment to improve protein adsorption and cell adhesion of smooth and rough titanium substrate [20]. 

Currently, the early loading of the dental implant has achieved a reduction of bone healing periods. In this study, we hypothesize that the increase of OH groups would be introduced on the SLA-treated surface by plasma treatments, and further improve the early-stage biocompatibility. Protein adsorption and cell adhesion are enhanced after the plasma treatments. According to prior evaluation, the cell responses of proliferation and differentiation are also promoted compared to the SLA-treated group. The SLA-treated surface via DC or RF plasma treatment was examined through morphology, hydrophilicity, and roughness. X-ray photoelectron spectroscopy (XPS) was used to examine chemical changes, especial in the functional OH group, due to plasma treatment. Then protein adsorption and MG63 cell behavior, including attachment, growth, and differentiation, on surfaces treated with DC and RF plasma, were investigated to evaluate biological responses. Finally, the effect of the functional OH group on the biological responses was examined. 

## 2. Results

### 2.1. Surface Analysis after Plasma Treatments

**Figure 1:**
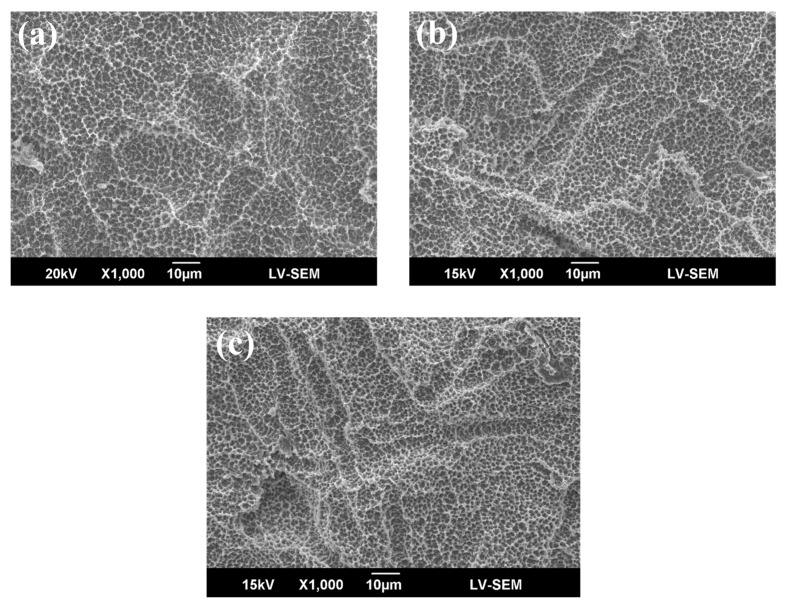
Typical SEM morphology of (**a**) control (SLA); (**b**) DC-50W; and (**c**) RF-200W. There are three representative SEM images because there is no topographical difference after plasma treatments (in seven groups), even in the highest power of Direct-Current (DC) and Radio-Frequency (RF) plasma treatments.

**Figure 2:**
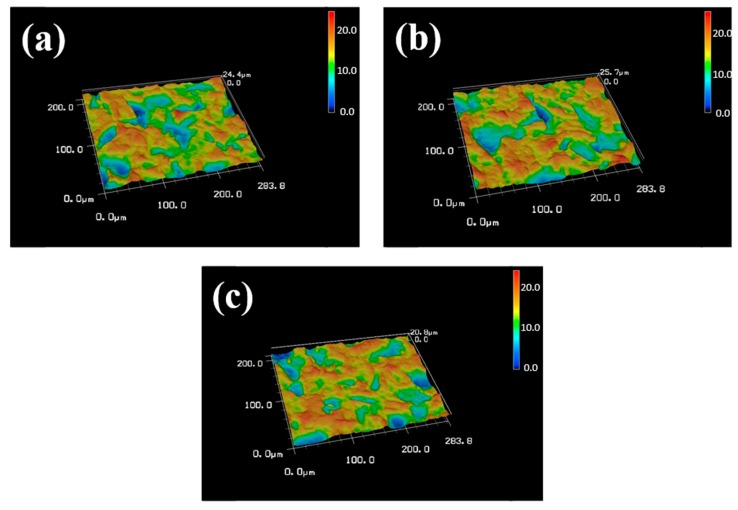
Typical morphology and surface roughness obtained using laser scanning microscopy of (**a**) control (Ra = 2.92 ± 0.22 μm); (**b**) DC-50W (Ra = 2.78 ± 0.25 μm); and (**c**) RF-200W (Ra = 2.65 ± 0.09 μm). (*n* = 3). Three representative images showed because there is no statistical difference, even in the highest power of DC and RF plasma treatments (in seven groups).

Figure 1 shows the sample SEM images with no treatment (control), DC plasma treatment, and RF plasma treatment. All SEM images show a similar surface structure, namely, a mountain and valley structure with micron and submicron scale notches. Figure 2 shows the 3D profile and roughness of the seven types of sample. The roughness ranged from 2.65 μm to 2.92 μm. No significant difference was found among these samples, as determined by ANOVA (*p* > 0.05). The roughness did not change even when the power of DC or RF plasma was increased. Neither DC nor RF plasma treatment changed the topography, morphology, or surface roughness. The samples became hydrophilic after DC or RF plasma treatment (data not shown).

XPS spectra results contain signals of O, F, C, and Ti, and traces of N on all surfaces. The DC- and RF-treated surfaces showed increased O/Ti compared to the control surface (Table 1). 

**Figure 3:**
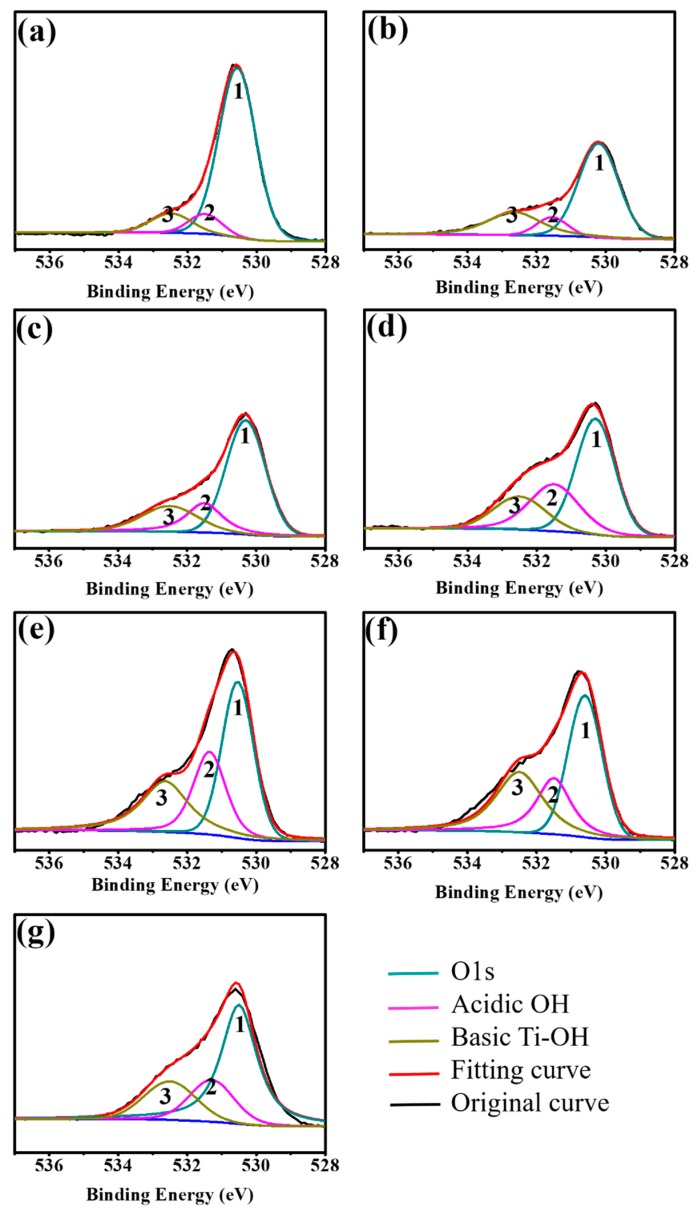
High-resolution O1s, basic Ti-OH, and acidic OH XPS spectra for surfaces subjected to DC and RF plasma treatments. (**a**) Control; (**b**) DC-30W; (**c**) DC-40W; (**d**) DC-50W; (**e**) RF-50W; (**f**) RF-100W; and (**g**) RF-200W.

The chemical states of O before and after DC, and RF oxygen plasma treatments, were detected by XPS and the corresponding O1s spectra. High-resolution XPS spectra for the O region of different samples are shown in Figure 3 and Table 2. 

The overlaps were resolved into their individual component using XPSPEAK 4.1 software (Version 4.1, The Chinese University of Hong Kong, Hong Kong). The peak at a binding energy of 530.2 eV corresponds to O1s, that at 531.5 eV corresponds to the acidic OH groups, and that at 532.5 eV corresponds to the basic Ti-OH groups [21]. The relative composition of O1s for DC plasma treatment samples changed with the power of DC oxygen plasma treatment, as shown in Table 2. The fraction of acidic bridged hydroxyl groups (acidic OH) increased from 8.21% to 9.86%, 18.89%, and 29.80% when the treatment power was increased from 0 W to 30 W, 40 W, and 50 W, respectively. In contrast, the fraction of O1s decreased from 81.79% to 64.16%, 60.28%, and 51.12%. The fraction of basic terminal hydroxyl groups (basic Ti-OH) did not regularly fluctuate when the treatment power was increased from 0 W to 50 W. The relative composition of O1s for RF plasma treatment samples changed with the power of RF oxygen plasma treatment, as shown in Table 2. The total fraction of basic Ti-OH increased when the treatment power was increased from 50 W to 200 W. We also observed that the fraction of O1s decreased from 52.39% to 44.02% and 43.09% when the treatment power was increased from 50 W to 100 W and 200 W, respectively. In this study, we emphasize that the OH groups including basic Ti-OH and acidic OH have an important influence on the biological response in the following sections. 

### 2.2. The Effect of Protein Adsorption

**Figure 4:**
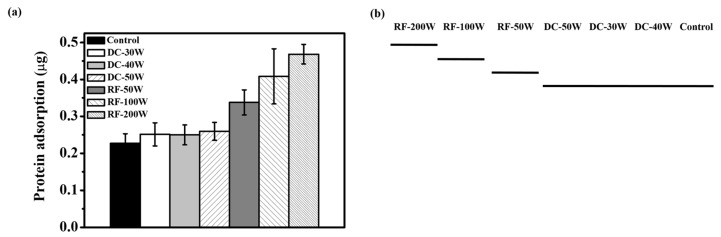
(**a**) Proteins adsorbed on control, DC-30W, DC-40W, DC-50W, RF-50W, RF-100W, and RF-200W (mean ± S.D., *n* = 4). Bars labeled with letters indicate significant difference between groups according to the Duncan test (*p* < 0.05). (**b**) Duncan grouping of protein adsorption

Figure 4 shows the protein adsorption on the seven types of sample (control, DC-30W, DC-40W, DC-50W, RF-50W, RF-100W, and RF-200W) after 1 h of incubation. The results of one-way ANOVA reveal that the group difference is statistically significant with a *p* value of less than 0.0001. The Duncan test was conducted at a significance level of 0.05. Figure 4a shows the results. In Figure 4b, the bold line beneath DC-50W, DC-30W, DC-40W, and the control indicates that their protein adsorptions showed no difference. The protein adsorptions of RF-200W, RF-100W, and RF-50W and that of each of DC-50W, DC-30W, DC-40W, and the control group are statistically different from each other. The protein adsorptions of the DC plasma treatment groups show no significant difference compared to the control. The RF plasma treatment groups are all statistically higher than those of the DC plasma treatment groups and the control.

### 2.3. The Influence of DC and RF Plasma Treatments on Cell Bioactivity

**Figure 5:**
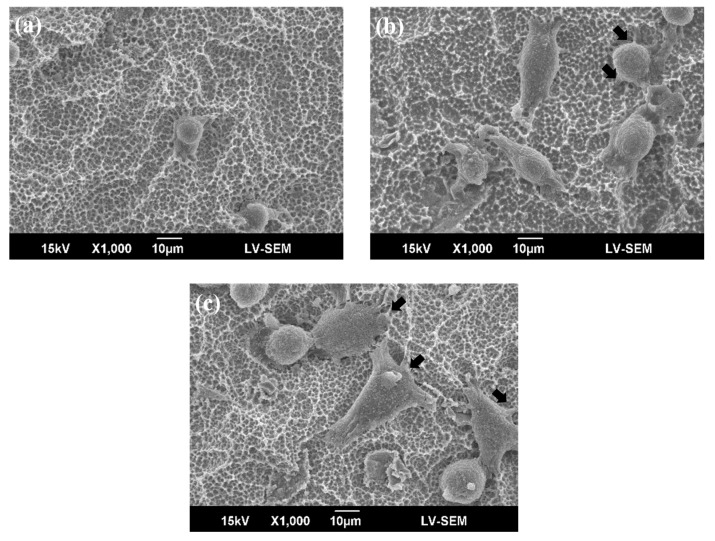
Representative morphology of MG63 cells cultured on (**a**) control; (**b**) DC-50W; and (**c**) RF-200W for 3 h. Filopodia are marked by black arrows. The filopodia are obviously observed in the highest power of DC and RF plasma treatments (in seven groups).

**Figure 6:**
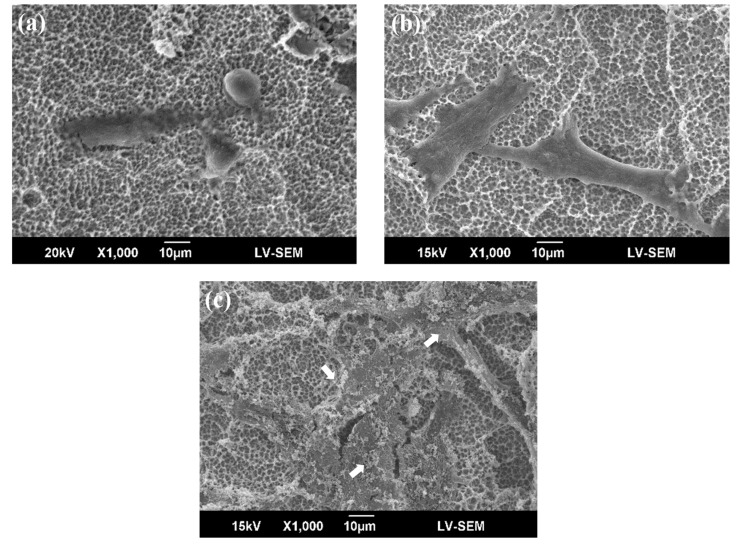
Representative morphology of MG63 cells cultured on (**a**) control; (**b**) DC-50W; and (**c**) RF-200W for 24 h. Extracellular matrix (ECM) components are marked by white arrows. The ECM only appeared abundantly on the highest power of RF plasma group (RF-200W).

Figure 5 and Figure 6 show morphological images of MG63 cells on DC- and RF-plasma-treated surfaces and the control surface after 3 h and 24 h of culture. The black arrows mark filopodia on the specimens, and the white arrows mark the ECM components. The cells had a spherical shape after 3 h for the control group. The DC treatment group had more filopodia than the control group. The RF treatment group had a flatter MG63 cell morphology than the DC plasma treatment and control groups. The RF plasma treatment groups showed apparent filopodia, especially in RF-200W groups. Thin filopodia were both easily observed for the DC and RF plasma treatment groups. After 24 h of culture, filopodia extended on the specimens, especially on DC- and RF-plasma-treated samples. The RF-200W groups had filopodia spread out from the cell body after 24 h. Cell adhesion on DC- and RF-plasma-treated samples was observed more on flattened shapes than control group. The RF-200W group had the most ECM-rich structures.

**Figure 7:**
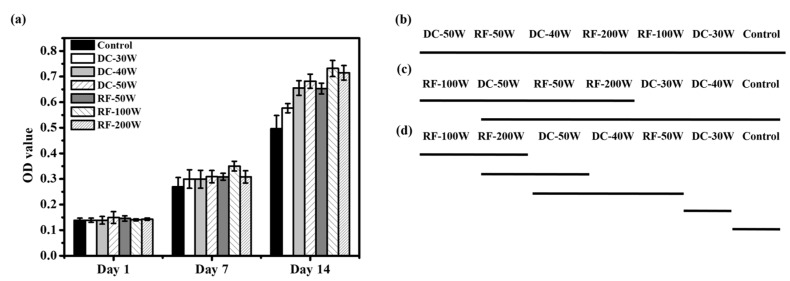
(**a**) Cell proliferation of MG63 cells cultured on control, DC-30W, DC-40W, DC-50W, RF-50W, RF-100W, and RF-200W for 1 day, 7 days, and 14 days (mean ± S.D., *n* = 5). Bars labeled with letters indicate significant difference between groups according to the Duncan test (*p* < 0.05). (**b**–**d**) Duncan grouping of cell proliferation,

The MG63 cell proliferation was evaluated using the alamarBlue assay after culture for 1 day, 7 days, and 14 days, respectively. Cell numbers were analyzed at 1 day, 7 days, and 14 days, reaching their peak at 14th day (Figure 7a). Figure 7b shows the Duncan test results for day 1. No significant differences were observed among the seven groups. After 7 days of culture, cell proliferation on the RF-100W group was the highest. The Duncan test results (Figure 7c) for day 7 show a bold line beneath RF-100W, DC-50W, RF-50W, and RF-200W, and another one beneath DC-50W, RF-50W, RF-200W, DC-30W, DC-40W, and the control. The Duncan test indicates that while the cell proliferation for RF-100W statistically differed from that for DC-30W, DC-40W, and the control, all other paired cell proliferation was considered statistically indistinguishable. At day 14, RF-100W had the highest cell proliferation. Figure 7d shows bold lines beneath (RF-100W, RF-200W), (RF-200W, DC-50W), and (DC-50W, DC-40W, RF-50W), denoting that the difference in cell proliferation between the paired specimens (RF-100W, RF-200W), (RF-200W, DC-50W), and (DC-50W, DC-40W, RF-50W) is statistically insignificant and that the differences among all other specimen pairs are statistically significant.

**Figure 8:**
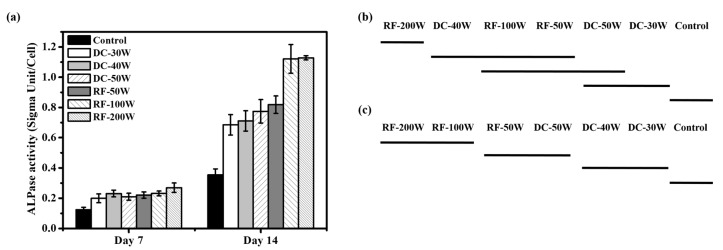
(**a**) Cell Differentiation of MG63 cells cultured on control, DC-30W, DC-40W, DC-50W, RF-50W, RF-100W, and RF-200W for 7 and 14 days (mean ± S.D., *n* = 4). Bars labeled with letters indicate significant difference between groups according to the Duncan test (*p* < 0.05). (**b**,**c**) Duncan grouping of ALPase activity,

Cell differentiation was measured from ALPase activity at day 7, and 14 respectively. After a culture period of 7 days, cells on RF-200W showed significantly higher differentiation than those for the DC plasma treatment and control groups. The Duncan test results (Figure 8b) for day 7 show a bold line beneath RF-200W, and another one beneath (DC-40W, RF-100W, RF-50W, DC-50W), (RF-100W, RF-50W, DC-50W), and (DC-50W, DC-30W). The difference in cell proliferation between the paired specimens (DC-40W, RF-100W, RF-50W, DC-50W), (RF-100W, RF-50W, DC-50W), and (DC-50W, DC-30W) is statistically insignificant and the differences among all other specimen pairs are statistically significant. After a culture period of 14 days, the MG63 cells cultured on RF-100W and RF-200W samples showed the highest differentiation. The Duncan test (Figure 8c) shows a bold line beneath (RF-200W, RF-100W) and another bold line beneath (RF-50W, DC-50W) and (DC-40W, DC-30W). The results show that the cell differentiation for RF-100W and RF-200W was significantly higher than that for the other groups. The cell differentiation with the two plasma treatments was higher than that of the control.

## 3. Discussion

In the past 50 years, implant dentistry for fully edentulous mandibles and maxillae has evolved from conventional delayed loading (3–6 months) to early loading (6–8 weeks) or immediate loading [9,10]. The breakthrough in loading protocol was achieved by early implant anchorage with surrounding bone tissue. The moderately rough surface of titanium implant plays a critical role in enhancing implant-to-bone interlocking. Surface treatments are also adapted to improve the biocompatibility of rough titanium implant. Upon implantation, initial interaction between host and implant involves the conditioning of the implant by serum and tissue fluids. The modified titanium could provide the active surface to attract more macromolecules, such as serum proteins, growth factors, and cytokines, and water on the surface of the implant [22]. These molecules will further influence the cell functions, such as attachment, spreading, migration, and differentiation, on surface of titanium. Some studies used surface treatments to modify the biocompatibility of SLA-treated titanium [23,24]. These treatments could produce hydrophilic surfaces and further improve biological responses. Except for wettability, we hypothesized that the amphoteric OH groups of oxide film on SLA-treated titanium are a key factor to biological responses. We used plasma treatment to modify the functional OH groups without variation of topography, and the relation between functional group and biological responses is confirmed in this study.

### 3.1. Surface Topography after Plasma Treatments

Previous investigations have reported that plasma treatment can change and even increase surface roughness [4,25]. For polymers, plasma bombards the polymer surface, increasing roughness [26,27]. Some relative studies proposed that plasma treatment might cause significant roughness changes on material surfaces, such as polished titanium, chitosan, or poly(etheretherketone) [4,28,29]. However, the plasma-treated SLA surface maintained its original surface texture, which contained micro- and sub-micro-scale roughness in all groups (as shown in Figure 1 and Figure 2). The topography of SLA-treated specimens didn’t change by the DC and RF plasma treatments. Notably, the rougher surface of titanium increased osteoblast-like cell attachment at 24 h [30]. Therefore, the maintenance of rough SLA surface preserves the bonding sites for bone cells. Lopez-Heredia et al. reported that RF plasma treatment (150W and 13.56 MHz) in N_2_, CO_2_, or N_2_/O_2_ atmosphere did not change the surface roughness of pure titanium after alkali treatment, and that RF plasma treatment enhanced bioactivity [19]. In this study, the plasma treatments maintained a micron and sub-micron scale topography of SLA specimens, which is consistent with previous results [19,20,21]. 

### 3.2. Plasma Treatments Enhance Hydrophilicity

After plasma treatment, the contact angles of all treated groups (DC-30W, DC-40W, DC-50W, RF-50W, RF-100W, and RF-200W) were too small to be detected, and that of the control was 109° (data not shown). The results show that both of DC and RF plasma treatments changed the hydrophobic surface to hydrophilic, which may enhance reactions such as protein adsorption and cell attachment, adhesion, proliferation, and even differentiation. Generally, hydrophilic surfaces exhibit better cell affinity, especially cell adhesion improvement [31]. A hydrophilic surface modification of titanium influenced differentiation and growth factor production for osteoblast-like cells [28,32]. Buser et al. examined the commonly used hydrophilic SLActive implant, which promoted more bone healing in the early stage than the traditional SLA method [23]. Therefore, hydrophilicity can be noted as a positive effect for titanium implant. Since the wettability of plasma-treated specimens is virtually the same, we have assumed that this factor does not influence biological responses in this study. 

### 3.3. Effect of Plasma Treatment on Functional OH Groups

The major difference between DC and RF plasma treatments is the power source. The ionization ratio or treatment power of DC and RF plasma affect the surface properties. In this study, treatment powers of 30 W, 40 W, and 50 W for DC plasma treatment and 50 W, 100 W, and 200 W for RF plasma treatment were chosen (Table 3). The DC plasma processing power is simple, but can only be applied to sputter metal, and for the sputtering insulator showed almost no function. RF plasma can be applied on metals, semiconductors, and insulators. RF power at a specific frequency of 13.56 MHz has been internationally allocated for RF power supplies. Because both of DC and RF plasma treatments have respective power range, we have selected two kinds of plasma treatments to modify SLA-treated specimens with different contents of functional OH groups. 

The basic Ti-OH and acidic OH groups on the SLA surface were abundant after treatment with DC or RF plasma (Table 2). The amphoteric OH content (basic Ti-OH and acidic OH groups) was higher in the DC and RF plasma treatment groups than the control group. For RF plasma samples, the basic Ti-OH and acidic OH groups were richer than those with DC plasma treatment. The XPS curve fitting after plasma treatment showed that the DC- and RF-plasma-treated samples increased the number of surface hydroxyl groups, including basic Ti-OH groups and acidic OH groups (Figure 3). This result is in agreement with the analysis by the DC and RF plasma treated of oxygen atmosphere for the SLA surface. After oxygen plasma treatment, the surface oxygen atomic concentrations (O/Ti) increased while the treating power of plasma treatments increased (Table 1). Basic Ti-OH also increased with plasma treatment power. Feng et al. indicated that compared to the acidic OH groups and the dispersion component of the total surface energy, the basic hydroxyl (basic Ti-OH) groups and the polar component are the most important factors between the osteoblast and titanium [30]. Hu et al. found that apatite precipitation was increased by the formation of basic Ti-OH and acidic OH groups on the surface of titanium discs using carbon dioxide plasma. Apatite precipitation increased due to the treatment attracting phosphate and calcium ions from the solution [21]. However, the relation between biological responses and contents of functional OH groups is not investigated in their studies. Tseng et al. used low-pressure RF oxygen plasma treatment on cp-Ti and Ti6Al4V surfaces to investigate the effect of plasma treatment time on cell response and found that 5 min was the best treatment time for cell behavior [28].

However, the relation between biological responses and contents of functional OH groups is also not investigated in Tseng et al.’s study. In addition, the mechanism of Ti-OH and hydrophilicity may cause the higher Ti-OH group to have higher surface polarity. Therefore, in our study we also found that H_2_O molecular can form H-bond with Ti-OH and further cause superhydrophilic surface [33].

### 3.4. Protein Adsorption of Plasma-Treated SLA Specimens

It was found that the contact angle of DC- and RF-plasma-treated SLA surfaces dramatically decreased. This phenomenon may increase surface energy and further improve cell adhesion and protein adsorption. Especially when the functional OH groups increased, the content of proteins adsorbed on SLA surfaces subjected to RF plasma treatment was much higher than those for the DC treatment and control groups (Figure 4). Recek et al. indicated that a large number of proteins can be absorbed on hydrophilic surfaces, leading to better cell proliferation and adhesion compared to hydrophobic surfaces [34]. The titanium rough surface with superhydrophilicity can cover with protein solution rapidly, and the rough property also increases protein contact areas [11]. Rupp et al. indicated that the higher free energy which correlated to superhydrophilicity can induce protein adsorption [35]. It is interesting that there is no statistical difference in protein adsorption between control and DC plasma-treated groups. The results indicated that the hydrophilicity is not the factor in determining the protein adsorption of SLA-treated specimens in this study. The wettability would depend on the contamination and functional OH group on the surface of titanium. The low-power DC-plasma treatment could clean and create low density of functional OH groups in this study. Both factors could not diminish the protein adsorption by factor of rough surface. The high-power RF-treated specimens have superhydrophilicity and higher density of amphoteric OH groups, and the content of protein adsorption shows higher than the other groups. It can be supposed that RF plasma treatment is an effective method to enhance protein adsorption of SLA specimens. 

### 3.5. DC and RF Plasma Treatments Promote Cell Responses

Despite the fact that surface roughness could influence cell attachment, the biomolecules in the culture medium could bind to functional OH groups on the specimen surface. The cell spreading and adhesion can be enhanced by binding to these molecules. Thus, the specimens treated with plasma treatment show much better cell spreading than untreated specimens. Furthermore, specimens treated with higher plasma power exhibit better cell spreading than specimens treated with lower plasma power. Circular cells appeared in the control and low-power groups. Filopodia extensions were easily observed in the high-power group after 24 h. According to the flattening and spreading of cells, lots of filopodia spread from the cell body, with a lower contact angle for a higher plasma power, after 48 h. The above results are similar to those in this study (Figure 5 and Figure 6).

**Figure 9:**
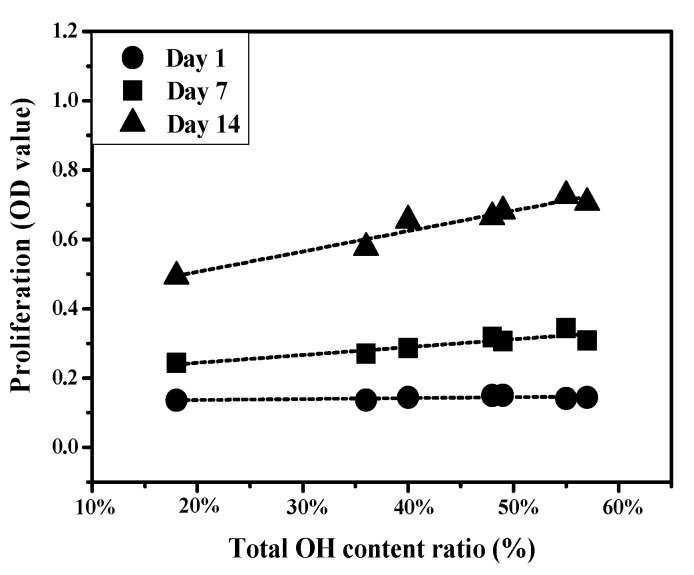
Relationship between cell proliferation and total amphoteric OH content.

**Figure 10:**
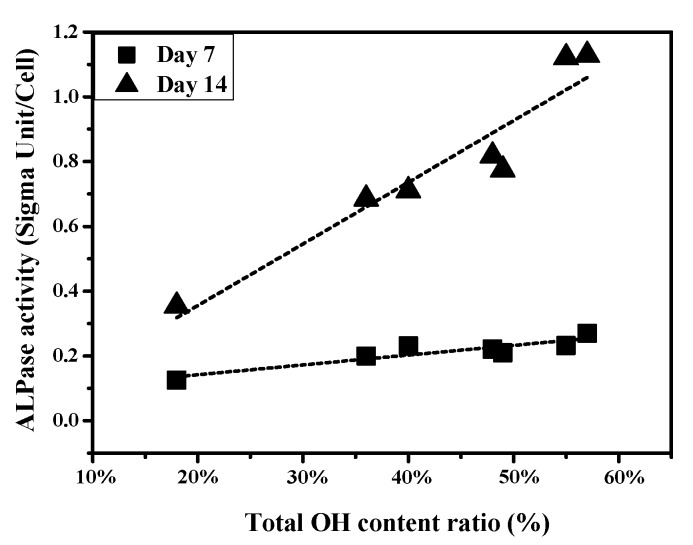
Relationship between cell differentiation and total amphoteric OH content.

Wang et al. confirmed that an increase in functional OH groups on a surface of polytetrafluoroethylene improves cellular activity [36]. Lee et al. found that the increased wettability of specimens treated with plasma led to better cellular attachment and then proliferation [37]. In the present study, plasma treatment promoted cell proliferation compared to control group. Figure 9 shows the relationship between cell proliferation and total amphoteric OH content. The content of amphoteric OH is positively related to cell proliferation on 14-day culture. The OH group influenced cell attachment. When the cell adhered to the plasma treated surface rapidly, the cell’s following reaction was started. Rapuano et al. reported that negatively charged oxides can lead to fibronectin adsorption and even the adsorption of other osteogenic proteins, enhancing osteoblast differentiation when RF plasma treatment is used [38]. As shown in Figure 8, the DC and RF plasma treatment groups exhibited high ALPase activity, which indicates higher cell differentiation than control group after 14 days. Figure 10 shows the relationship between ALPase activity and total amphoteric OH. The content of functional OH groups is positively correlated to ALPase activity after 7-day and 14-day cultures. The high contents of functional OH groups improved proliferation and differentiation of osteoblast cells. Therefore, we suggest that content of functional OH group will mainly influence biological responses of SLA specimens more than hydrophilicity in the present study.

In clinical scenario, the dental implant should achieve osseointegration before functional loading. The guidelines suggest that dental implant needs sufficient primary stability and a healing period of 3–6 months without functional loading [39]. Recently, dental implant has evolved from conventional delayed loading to early loading or immediate loading. The bioactive surface of titanium implant could increase bone healing rate and achieve early loading. Previous studies demonstrated that hydrophilic surface could provide protein adsorption and cell attachment after implantation. Different methods, such as low pressure plasma treatment, atmosphere plasma treatment, and ultraviolet (UV) light treatment, have been adapted to increase wettability of titanium implant. Although hydrophilic specimens could present better protein and cell responses, the hydrophilicity could not totally explain the mechanisms for biocompatibility of titanium. In general, the oxide film of titanium may play an important role in biological responses. The oxide film could present two types of functional OH groups to simultaneously act as anion and cation exchange sites. In the present study, the factor functional OH groups are better than hydrophilicity at explaining the protein and cell responses of SLA-treated specimens. Overall, the limitation of this study is that the OH groups may decay over time or be influenced by surrounding environment. Fortunately, there are lots of solutions for preserving the surface properties, such as keeping the specimen in isotonic NaCl solution [40,41]. In this study, the plasma treated surface all showed more functional OH groups, and the power increase causes the excellent biological response. However, further in vivo experiments should be carried out to investigate the relation between functional OH group and biocompatibility of SLA-treated titanium implant. The plasma treatments are simple and using rapid methods to activate SLA implant surface, and this method shows the potential applied in early-stage bone healing of dental and orthopedic implants.

## 4. Materials and Methods

### 4.1. Sample Preparation

Commercially pure titanium discs (cpTi, Grade II, ASTM F67, Taiwan) measuring 12.7 mm in diameter, 2 mm in thickness, and total discs number of 224 were used. The surface of samples was grinded with emery paper and cleaned in acetone using an ultrasonic machine, ethanol, and distilled water, each for 15 min, and then dried in an oven. The above discs were sand-blasted with large grit Al_2_O_3_ (white alumina with a particle size of 355–425 μm) at 4 kg/cm^2^ and subsequently etched with a mixed acid containing a volume ratio of HCl/H_2_SO_4_ of 1/3 for 30 min at 80 °C, followed by washing in acetone, ethanol, and distilled water. The obtained SLA samples were kept in vacuum conditions until use. The experiments and examination are all randomized.

### 4.2. Plasma Treatments

The SLA samples were separately surface-modified via plasma treatment under an oxygen atmosphere. The DC plasma equipment (home-made equipment) consists of plasma DC power supply, react chamber, gas injection cylinder, and evacuation system. The samples were put into the middle of the react chamber, the working area diameter was 12 cm, and the distance between the electrodes was 6 cm. The conditions of plasma power, gas flow rate, and reacting time are controlled, and in this study we use power of 30 W, 40 W, and 50 W, gas flow rate of 20 sccm/min, and the constant reacting time of 5 min. The chamber was first evacuated to less than 5 × 10^−2^ Torr before being filled with oxygen gas. After the chamber pressure had stabilized to 2 × 10^−1^ Torr, glow discharge plasma was produced by controlling the electrical power at 30 W, 40 W, or 50 W for 5 min. The gas flow rate was controlled at 20 sccm/min using a flow meter. Finally, the plasma-treated specimens were further exposed to the oxygen atmosphere for another 10 min and then put into absolute alcohol before being analyzed. 

The RF plasma equipment (home-made equipment) consists of a load lock transfer arm, react chamber, gas injection cylinder, and evacuation system. The load lock arm contains a sample holder (working area is 15 cm^2^) that can hold samples and a mechanical transfer mechanism to move samples to and from the process chamber. The distance between the electrodes was 8 cm and the sample transfer was into the chamber at the middle of the electrodes. For RF plasma treatment, the SLA samples were placed on a clean tray (load lock arm), which was inserted into the RF unit. The unit was placed under dry vacuum. When the vacuum was less than 2 × 10^−2^ Torr, oxygen slowly entered the system via a needle valve. The gas flow rate was controlled at 20 sccm/min using a flow meter. When the chamber pressure had stabilized to 1.3 × 10^−1^ Torr, the operating conditions were 50 W, 100 W, or 200 W at 13.56 MHz for 5 min. The sample nomenclature is as follows: control (only SLA), DC-30W (SLA-treated titanium subjected to DC plasma at 30 W), DC-40W (SLA-treated titanium subjected to DC plasma at 40 W), DC-50W (SLA-treated titanium subjected to DC plasma at 50 W), RF-50W (SLA-treated titanium subjected to RF plasma at 50 W), RF-100W (SLA-treated titanium subjected to RF plasma at 100 W), and RF-200W (SLA-treated titanium subjected to RF plasma at 200 W) (see Table 1). All samples were kept in absolute alcohol prior to use.

### 4.3. Specimen Characterization

The surface roughness and three-dimensional (3D) profile images were obtained using laser scanning microscopy (VK-X200; Keyence, Taiwan). The laser beam emitted from the laser light source scans the target surface and gets the roughness average (Ra), which is the arithmetic average of the absolute values of the profile heights over the evaluation length. The surface morphology was analyzed using scanning electron microscopy (SEM; JSM-6390LV, JEOL, Tokyo, Japan). The surface composition was determined using XPS (PHI Quantera SXM, ULVAC-PHI, Kanagawa, Japan) with a monochromatic Al Kα (hν = 1486.6 eV) source. A 45° takeoff angle was used. The deconvolution of the high-resolution spectrum was performed by the Gauss-Lorentz function (XPSPEAK). An element was quantified based on the peak area of the sensitivity factor adjusted by the instrument manufacturer. 

### 4.4. Protein Adsorption

Bovine serum albumin (BSA; Sigma-Aldrich, purity 99.8%, St. Louis, MO, USA) was dissolved in phosphate-buffered saline (PBS) buffer at pH 7.4. The concentration of BSA was 1 mg/mL. The seven types of sample were incubated for 1 h at 37 °C in the protein solution and then removed and rinsed with distilled water to remove excess protein solution. The bicinchoninic acid (BCA; Thermo Fisher, Waltham, MA, USA) assay was used to evaluate the protein concentration of the seven types of sample. An enzyme-linked immunosorbent assay (ELISA) plate reader (Tecan, Sunrise, Zürich, Switzerland) at 562 nm was used for analysis. The specimen number of each group is four for protein adsorption.

### 4.5. Cell Culture

Human osteoblast cells, isolated from human osteosarcoma (MG63, ATCC number: CRL-1427, USA), were purchased from the American Type Culture Collection (ATCC, Rockville, MD, USA). Dulbecco’s modified Eagle’s medium (DMEM; Gibco, Gaithersburg, MD, USA) was used and supplemented with 10% fetal bovine serum (FBS; Gibco, Gaithersburg, MD, USA) for the MG63 cells. The MG63 cells were grown at 37 °C in a 5% CO_2_ atmosphere. Fresh medium was added every other day until harvesting. All specimens were placed in a 24-well plate (Nunclon Nalge Nunc Int., Roskilde, Denmark) and sterilized in 70% alcohol for 1 h.

### 4.6. Cell Morphology

The MG63 cells were cultured on sterilized specimens at a density of 5 × 10^3^ cells/cm^2^ for 3 h and 24 h. At the harvest points of 3 h and 24 h, the medium was removed and washed with PBS. The specimens were fixed with 2.5% glutaraldehyde overnight. The specimens were washed with PBS, and then immersed in 1% tannic acid for 1 h. Then, the specimens were rinsed with PBS again, and dehydrated sequentially in 30%, 50%, 75%, and 100% ethanol for 10 min. Finally, they were soaked in hexamethyldisilazane (HMDS; Sigma, St. Louis, MO, USA) for 30 min. Gold was coated onto the specimens to let the cells be conductive using a sputter coater, and the specimens were observed using SEM.

### 4.7. Cell Proliferation

The proliferation rate was examined with a cell density of 5 × 10^3^ cells/cm^2^ at 1 day, 7 days, and 14 days of culture. After the harvesting of MG63 cells, 10% alamarBlue (Thermo Fisher, Waltham, MA, USA) was added to each of the 24 wells as the working solution for 3 h at 37 °C. The harvested working solutions were examined using an ELISA plate reader at a wavelength of 570 nm against a reference wavelength of 600 nm. The sample number is five for each group.

### 4.8. Cell Differentiation

Alkaline phosphatase (ALPase) is a marker representing osteoblast differentiation at the early stage, and can cause the following differentiation responses, especially bone formation. The sterilized specimens were placed in a 24-well plate seeded at a cell density of 5 × 10^3^ cells/cm^2^. At the harvest time on days 7 and 14, SIGMAFAST™ (Sigma, St. Louis, MO, USA) ρ-nitrophenyl phosphate (ρNPP) tablets were used to evaluate ALPase activity. A lysis buffer (containing 0.1% triton-X-100) was used to break the cell membrane using a cyclic freezing/thawing process. After 100 μL of ρNPP working solution was added to each well for 30 min at 37 °C, 0.05 M NaOH was added to stop the reaction. Then, the working solution was taken out for analysis using an ELISA plate reader at 405 nm. The sample number of each group is four for cell differentiation.

### 4.9. Statistical Analysis

The surface roughness, protein adsorption, and cell proliferation and differentiation data are shown as the mean ± standard deviation (SD). One-way analysis of variance (ANOVA) was employed to determine significant differences between control, DC plasma treatment, and RF plasma treatment groups. The Statistical Analysis System (SAS, SAS Institute Inc., Cary, NC, USA) was used to analyze the data. Statistical significance was regarded as *p*-value < 0.05. The Duncan test was performed for a significance level of 0.05 to indicate treatment effects.

## 5. Conclusions

The rough specimens treated with plasma exhibit superhydrophilicity and a higher surface O/Ti ratio. After plasma treatment, the increase of functional OH groups can promote protein adsorption and biological responses. The SLA-treated rough surface combined with RF plasma treatment is an excellent surface modification for clinical application.

## Figures and Tables

**Table 1:** Surface chemical composition (atomic %) obtained from X-ray photoelectron spectroscopy (XPS) analyses for control, DC plasma treatment, and RF plasma treatment groups.

	O1s	F1s	C1s	Ti2p	N1s	O/Ti
Control	57.4	―	19.6	21.5	1.5	2.6
DC-30W	58.8	1.7	16.8	22.5	0.2	2.6
DC-40W	59.5	1.8	17.9	20.3	0.5	2.9
DC-50W	57.0	5.1	18.8	18.9	0.2	3.0
RF-50W	55.7	5.8	19.0	19.2	0.3	2.9
RF-100W	51.5	4.4	26.9	15.8	1.4	3.3
RF-200W	47.4	2.7	36.0	13.0	0.9	3.7

**Table 2:** Percentages of O1s, acidic OH, and basic Ti-OH groups obtained by decomposing oxygen XPS spectra for control, DC plasma treatment, and RF plasma treatment groups.

	Basic Ti-OH	Acidic OH	O1s
Control	10.00	8.21	81.79
DC-30W	25.98	9.86	64.16
DC-40W	20.83	18.89	60.28
DC-50W	19.08	29.80	51.12
RF-50W	21.91	25.70	52.39
RF-100W	28.49	27.49	44.02
RF-200W	33.05	23.86	43.09

**Table 3:** Parameters of control, DC plasma treatment, and RF plasma treatment groups. All specimens were pretreated using SLA method. Control group had no additional treatment. Power values of DC plasma were 30 W, 40 W, and 50 W. Power values of RF plasma were 50 W, 100 W, and 200 W.

	Surface Pretreatment	Pressure	Flow Rate	Power	Nomenclature
**Control**	SLA	N/A	N/A	N/A	SLA
**DC plasma**				30 W	DC-30W
	SLA	2 × 10^−1^ Torr	20 sccm	40 W	DC-40W
				50 W	DC-50W
**RF plasma**				50 W	RF-50W
	SLA	1.3 × 10^−1^ Torr	20 sccm	100 W	RF-100W
				200 W	RF-200W
